# Outcomes in Bone Giant Cell Tumors Treated With Surgical Resection With and Without Denosumab Injection: A Single-Institution Retrospective Study

**DOI:** 10.7759/cureus.26869

**Published:** 2022-07-14

**Authors:** Ali H AlYami, Abdulaziz Nazer, Hussam H Bashawieh, Albara A Dabroom, Majd Saem Aldahar, AlWaleed A AlYami, Bandar N AlMaeen

**Affiliations:** 1 Department of Surgery, Ministry of the National Guard – Health Affairs, Jeddah, SAU; 2 Department of Surgery, King Abdullah International Medical Research Center, Jeddah, SAU; 3 Department of Surgery, King Saud Bin Abdulaziz University for Health Sciences, Jeddah, SAU; 4 Department of Medicine, College of Medicine, King Saud Bin Abdulaziz University for Health Sciences, Jeddah, SAU; 5 Department of Orthopaedics, King Saud Bin Abdulaziz University for Health Sciences, Jeddah, SAU; 6 Department of Surgery, College of Medicine, Jouf University, Al-Jouf, SAU; 7 Department of Orthopedic Surgery, King Faisal Specialist Hospital & Research Centre, Riyadh, SAU

**Keywords:** surgical resection, saudi arabia, recurrence rate, denosumab, giant cell tumor of the bone

## Abstract

Background: Giant cell tumors of the bone (GCTB) are rare, benign, aggressive, recurrent tumors that are most often found at the ends of long bones. They account for 5% of all primary bone tumors and 20% of all benign bone tumors. The clinical features of GCTB include local swelling, pain, and limitations in joint movement. Approximately half of GCTB arise around the knee joint, affecting either the distal femur or proximal tibia. Tissue biopsy reveals an excess of multinucleated giant cells on a stromal cell background, indicating a diagnosis. Intralesional curettage is used to treat GCTB and is associated with minimal disability; however, local recurrence may occur in many patients. Resection and endoprosthetic repair or bone graft reconstruction are often used to treat GCTB near the joint. To our knowledge, there are currently no studies on this topic in the city of Jeddah, where we conducted our study. Our aim was to evaluate the outcome of surgical resection accompanied by denosumab injection compared to that of surgery alone in treating GCTB.

Methods: All cases of GCTB at King Abdulaziz Medical City, Jeddah, between January 2008 and December 2018, that fulfilled the inclusion and exclusion criteria were included. All cases of GCTB in the pre-specified period were classified as surgical resection with denosumab injection or surgical resection alone. The outcomes of the two modalities were compared. Recurrence was investigated in patients belonging to both the groups.

Results: Twenty-six cases that met the inclusion criteria were included in the study and the data were analyzed. The subjects were divided into two groups: denosumab and surgery (n = 7) and surgery alone (n = 19). Patients treated with denosumab and surgery had a higher recurrence rate (57%); however, the difference was not significant (p = 0.407).

Conclusion: Our study showed that when comparing local recurrence after curettage in patients treated with denosumab and patients who did not receive it, preoperative denosumab therapy was associated with an increased incidence of local recurrence. We recommend a systematic review that can include more studies in this field to acquire more definitive results regarding this topic.

## Introduction

Giant cell tumors of the bone (GCTB) are rare, benign, aggressive, recurrent tumors [1]. They are most often found at the ends of long bones and account for 5% of all primary bone tumors and 20% of all benign bone tumors [2]. The GCTB incidence is 1.3 per million persons per year based on a population study conducted in Sweden, which reported a female predominance with a female to male ratio of 1.3-1.5:1 [3]. GCTB emerge following skeletal maturity, with a peak incidence among those in their 20s and 30s [4]. The clinical features include swelling, pain, and limitations in joint movement [3]. Around half of GCTB arise around the knee joint, either affecting the distal femur or proximal tibia [1].

Tissue biopsy reveals an excess of multinucleated giant cells on a stromal cell background, indicating a diagnosis. These neoplastic stromal cells highly express receptor activator of the nuclear factor-kappa ß (RANK) ligand (RANKL) and induce receptor activation of RANK-positive osteoclast-like giant cells. The RANK-RANKL interaction plays a significant role in bone resorption. Intralesional curettage is used to treat GCTB and is associated with minimal disability; however, local recurrence may occur in many patients [5].

Resection and endoprosthetic repair or bone graft reconstruction are often used to treat GCTB near the joint. However, they are also associated with a significant degree of impairment. The rates of local recurrence after curettage or resection have been reported to be 16%-50% and 0%-12%, respectively [6]. Denosumab is a human monoclonal antibody that exerts its effects by binding to the RANKL, thereby interrupting the RANK-RANKL interaction, which is thought to be essential for osteoclast activation and differentiation. As a result, it inhibits bone destruction and eliminates giant cells [5].

In 2013, denosumab was approved by the US Food and Drug Administration to treat adults and skeletally mature adolescents with unresectable GCTB or patients for whom resection would result in extreme morbidity. In a study, it was found that preoperative denosumab therapy resulted in surgical downstaging in patients with operable giant cell tumors (GCTs) of the bone [7]. In another study, all patients who received denosumab had tumor control, while 40% of those who stopped taking denosumab had tumor growth after a median of eight months [8]. Conversely, Traub et al. stated that local tumors recurred in 15% of patients following preoperative denosumab therapy and curettage after a median of 30 months of follow-up [9].

A systematic review by Luengo-Alonso et al. included 19 studies with 1095 patients and concluded that‏ denosumab demonstrated a strong but unpredictable histological response when used as adjuvant therapy for GCTB. However, denosumab use is controversial as it does not show any effect on local recurrence [10]. According to a study by Urakawa et al. in Japan, which assessed the clinical outcome of GCTs after curettage with and without perioperative denosumab, the risk of recurrence was still high in both arms, especially in grade III Campanacci [11].

There are contradictory studies on whether preoperative denosumab treatment of GCTB raises the risk of local recurrence in patients treated with curettage. Moreover, to our knowledge, there are currently no studies on this topic in our area. Our aim was to evaluate the surgical resection outcome accompanied by denosumab injection compared to surgery alone, in treating bone GCTs.

## Materials and methods

This retrospective cohort study included all cases of GCTB treated at our institution (King Abdulaziz Medical City, Jeddah, or KAMC-J) between January 2008 and December 2018. Patients were considered eligible if they had histologically confirmed GCTB. This study was approved by the Institutional Review Board of the King Abdullah International Medical Research Center, Jeddah (approval #SP20/236/J); informed consent was taken from all patients.

Data for patient demographics and different treatment modalities, including denosumab, response rate, disease progression, and survival data, were collected. A data collection sheet was used to collect the demographic variables of the patients, including age, gender, smoking status, mobility, body mass index (BMI), duration from diagnosis to surgery (days), age at diagnosis, presenting symptoms, site of the tumor, type of surgery, and recurrence of the lesion, as well as the status at the last presentation, for both denosumab and surgery and surgery alone groups. Denosumab has been approved in our institution as a standard of care in the management of patients with advanced GCTB since 2014. The schedule used to date in our routine practice is denosumab 120 mg subcutaneously every 28 days, with two additional loading doses on days 8 and 15 of the first month. The sample size included 26 cases of GCTB diagnosed between 2008 and 2018 at KAMC-J; patients were divided into two groups: denosumab and surgery (n = 7) and surgery alone (n = 19). Response was assessed retrospectively according to the recorded clinical and radiological evaluation, including the presence of pain and size of the lesion.

All data were acquired from the Best Care System using electronic medical records at King Abdulaziz Medical City. However, a non-probability (inclusive) consecutive sampling technique was used to select all the patients who met the inclusion and exclusion criteria. The subjects were divided into two groups: denosumab and surgery (n = 7) and surgery alone (n = 19). Data were collected by the co-authors of the study.

Data were verified, coded by the researcher, and analyzed using IBM SPSS 24.0 (IBM Corp., Armonk, NY). Categorical variables were reported as percentages, and numerical variables were reported as means or medians. Normally distributed numerical variables were reported using the mean and standard deviation (SD), and skewed distributed variables were reported using the median and interquartile range (IQR). Categorical variables were compared using the chi-square test and Fisher’s exact test. Statistical significance was set at p < 0.05.

## Results

Among all cases diagnosed with GCTB, 26 patients met the inclusion criteria and were thus included. They were divided into two groups: first group, denosumab and surgery (n = 7), and second group, surgery alone (n = 19). There were 5 (71%) male patients in the first group and 13 (68%) in the second group. Smoking was not prevalent in the study as only 1 (14%) case was present in the first group and 3 (16%) cases were found in the second group (Table 1).

**Table 1:** General demographics *Fisher’s exact test

Variables	Categories	Denosumab and surgery		Surgery only		p-value
		N	Total %	N	Total %	
Gender	Male	5	71	13	68	1*
	Female	2	29	6	32	
Smoking	Yes	1	14	3	16	1*

The study sample had a mean BMI of 25.7 (SD = 5.3) in the first group and 23.4 (SD = 5.5) in the second group. The median age at diagnosis was 36.5 years (SD = 13.1) in the first group and 28.2 years (SD = 13.8) in the second group (Table 2).

**Table 2:** Demographic characteristics

Categories	Denosumab and surgery			Surgery only		
	N	Mean	SD	N	Mean	SD
BMI	7	25.7	5.3	19	23.4	5.5
Age at the diagnosis (years)	7	36.5	13.1	19	28.2	13.8
Duration since diagnosis to surgery (days)	7	302	241	19	64	105

Numerous signs and symptoms were present at diagnosis, of which pain was the most common symptom in the first and second groups (100% and 68.4%, respectively). However, pathological fracture was the least common presentation, as only 1 (14%) case suffered from pathological fracture in the first group and 5 (26%) were found in the second group. Seven different sites were recognized in this sample, and proximal tibia was the most common tumor site as seen in 28.6% cases in the first group and 42.1% in the second group. Detailed data on the clinical manifestations and other features are shown in Table 3.

**Table 3:** Clinical manifestation at presentation and tumor features *Fisher’s exact test **Pearson’s chi-square test

Variables	Categories	Denosumab and surgery		Surgery only		p-value
		N = 7	Total %	N = 19	Total %	
Presenting symptoms	Pain	7	100	13	68.4	0.146*
	Swelling	4	57	14	74	0.635*
	Limitation of activity	1	14	5	26	1*
	Pathological fracture	1	14	5	26	1*
Site of the tumor	Distal radius	2	28.6	2	10.5	0.309**
	Proximal fibula	2	28.6	0	0	
	Distal femur	0	0	7	36.8	
	Proximal tibia	2	28.6	8	42.1	
	Distal fibula	0	0	1	5.3	
	First metacarpal	1	14.3	0	0	
	Ulna	0	0	1	1.9	

Most of the cases (65.2%) were treated with curettage and bone graft: 5 (71.4%) in the first group and 13 (68.4%) in the second group. Only one case from the second group underwent resection and arthrodesis. Detailed data are shown in Table 4.

**Table 4:** First-time treatment data *Pearson’s chi-square test

Variables	Categories	Denosumab and surgery		Surgery only		p-value
		N = 7	Total %	N = 19	Total %	
Type of surgery	Curettage and bone graft	5	71.4	13	68.4	0.825*
	Curettage and bone graft and cementation	2	28.6	5	26.3	
	Resection and arthrodesis	0	0	1	1.9	

Recurrence was investigated in patients in both the groups. Patients treated with denosumab and surgery had a higher recurrence rate (57%); however, the difference was not significant (p = 0.407) as shown in Table 5.

**Table 5:** Association between recurrence and treatment *Fisher’s exact test

Variables	Categories	Denosumab and surgery		Surgery only		p-value
		N	Total %	N	Total %	
Recurrence	Yes	4	57	7	37	0.407*
	No	3	43	12	63	

In the last follow-up with a minimum of three years, most patients improved clinically, as 4 (57.1%) from the first group and 15 (78.9%) from the second group were alive and asymptomatic. Detailed data are shown in Table 6.

**Table 6:** Status at the last presentation

Variable	Categories	Denosumab and surgery		Surgery only	
		N	Total %	N	Total %
Status at the last presentation	Alive and asymptomatic	4	57.1	15	78.9
	Alive but symptomatic	2	26.6	4	21.1
	Dead	1	14.3	0	0

## Discussion

GCTB are rare benign bone tumors that most commonly occur in the meta-epiphyseal region of long bones [12]. They account for approximately 5%-6% of all primary bone tumors [13,14]. This tumor is usually benign in nature but can be aggressive and may affect the mobility and functionality of the nearby joints, which can also be attributed to the osteolytic nature of the tumor [12]. GCTB consist of three cardinal components: mononuclear spindle-shaped stromal cells, multinucleated osteoclast-like giant cells that are eventually responsible for excessive bone resorption, and mononuclear cells of monocyte lineage [14]. Surgery, either curettage or en bloc resection, with or without adjuvants, is the mainstay treatment for this tumor [12].

In this study, 26 cases were included, of which 70% involved men, and the median age at diagnosis was 28 years, which is consistent with the results of a meta-analysis conducted by Yayan, in which the most commonly reported age of GCTB onset was the third decade of life, specifically 30 years, followed by the second decade [15].

Regarding the presentation and clinical manifestations at the time of diagnosis, pain was the most commonly reported symptom (76.9%), followed by swelling (69.2%), whereas stiffness was reported in only one case, making it the least common. Similarly, pain and swelling were the typically reported symptom and sign at presentation in another study [16]. Additionally, Martin and McCarthy reported that all 10 cases of GCTB of the sacrum and most cases of GCTB of the spine complained of pain at the time of diagnosis [17]. The proximal tibia was the most common site of the tumor in this study, followed by the distal femur and radius, which is consistent with the findings in the literature [16,18]. According to a retrospective study involving 230 patients with biopsy-proven GCTB between 1980 and 2010, the most common location was the distal femur (n = 72), followed by the proximal tibia (n = 48) and distal radius (n = 25) [19].

The majority of the cases in this study were treated with curettage and bone grafting (65.2%), while seven of the cases underwent curettage, bone grafting and cementation, and one case required resection and arthrodesis. This is consistent with the literature, as curettage was found to be the most common type of treatment used [15]. Denosumab was used in seven cases for a median duration of six months, and this medication works by binding and inhibiting the RANKL, which in turn results in the reduction of formation and activation of osteoclasts. As a result, bone resorption decreases, leading to fewer fractures and other bone complications [15]. The response to denosumab was determined based on computed tomography or magnetic resonance imaging performed at the six-month follow-up [15]. A complete response is defined as having no evidence of GCTB in imaging; partial response as a reduction of at least 30% in the tumor diameter; stable disease, the presence of an unchanged tumor size; and progressive disease, when there is an increase in the tumor size by 20% [15].

Unfortunately, 11 patients had a recurrence of the disease within a median duration of 286.5 days after treatment, and the most commonly performed surgeries in these recurrent cases were curettage and bone grafting. Denosumab was added in 7 of 11 patients who had a recurrence since they had a more advanced and complicated course of the disease. This could explain why denosumab is associated more with recurrence since it is usually used in advanced cases. According to Kremen et al., in their retrospective cohort study, 21 of the 216 included cases had local recurrence with a median time since treatment of 20 months [19]. The local recurrence-free survival for patients with lesions treated with curettage in the aforementioned study was 84% at 122 months, while it was 96% at 127 months for those treated with resection.

A variety of factors in this study were investigated, such as age, gender, use of denosumab, number of cycles of treatment, and type of surgery performed, to assess their effects on the recurrence rate of the disease. The only variable in this study that showed a statistically significant association with recurrence rate was the presence of pathological fractures. This was further supported by O’Donnell et al., who reported that pathological fractures increase the risk of recurrence [20]. Furthermore, denosumab use was associated with a slightly higher recurrence rate (57%) compared to cases that did not receive denosumab (37%); however, this was not statistically significant.

In a systematic review comparing local recurrence after curettage in patients treated with denosumab and those without, it was observed that preoperative denosumab therapy was associated with an increased incidence of local recurrence [5]. This study also had limitations; first, the sample size included in this study was small. Second, this study was retrospective in nature. However, this study also has merits, one of which is offering a guide on how to successfully manage a case of a giant cell tumor. It was also found that the rate of local recurrence rate was increased when denosumab was added to the treatment plan.

## Conclusions

Our study showed that when comparing local recurrence after surgery in patients who underwent denosumab therapy versus those who did not, denosumab therapy was found to be associated with an increased incidence of local recurrence of GCTB. We recommend a systematic review that can include more studies in this field to acquire more definitive results regarding this topic.
